# The virus–receptor interaction in the replication of feline immunodeficiency virus (FIV)^☆^

**DOI:** 10.1016/j.coviro.2013.08.003

**Published:** 2013-12

**Authors:** Brian J Willett, Margaret J Hosie

**Affiliations:** MRC-University of Glasgow Centre for Virus Research, Glasgow, United Kingdom

## Abstract

The feline and human immunodeficiency viruses (FIV and HIV) target helper T cells selectively, and in doing so they induce a profound immune dysfunction. The primary determinant of HIV cell tropism is the expression pattern of the primary viral receptor CD4 and co-receptor(s), such as CXCR4 and CCR5. FIV employs a distinct strategy to target helper T cells; a high affinity interaction with CD134 (OX40) is followed by binding of the virus to its sole co-receptor, CXCR4. Recent studies have demonstrated that the way in which FIV interacts with its primary receptor, CD134, alters as infection progresses, changing the cell tropism of the virus. This review examines the contribution of the virus–receptor interaction to replication *in vivo* as well as the significance of these findings to the development of vaccines and therapeutics.

## Highlights

• Expression of viral receptor CD134 is consistent with FIV cell tropism.
• Differential usage of CD134 by individual strains of FIV defined by requirement for CRD2 of CD134.
• CRD2-dependent strains dominate in early infection.
• CRD2-independent strains emerge in late infection.
• Selective expansion of CRD2-dependent variants following experimental transmission.

## Graphical abstract

**Figure:**
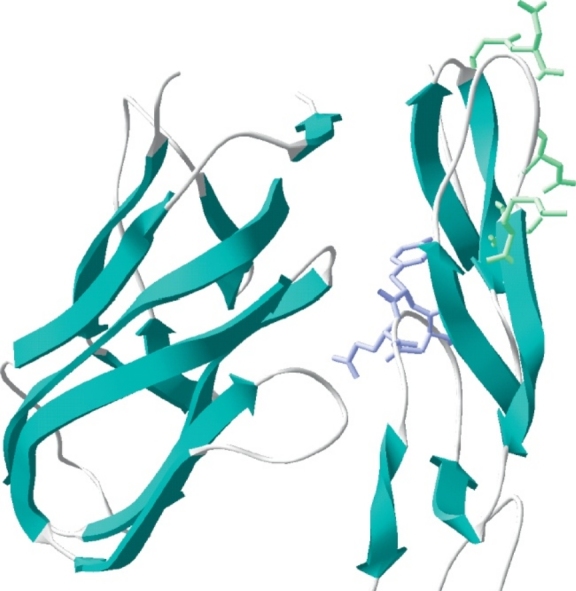

**Current Opinion in Virlogy** 2013, **3**:670–675

This review comes from a themed issue on **Virus replication in animals and plants**

Edited by **Ben Berkhout** and **Kuan-Teh Jeang**

For a complete overview see the Issue and the Editorial

Available online 28th August 2013

1879-6257/$ – see front matter, © 2013 The Authors. Published by Elsevier B.V. All rights reserved.

**http://dx.doi.org/10.1016/j.coviro.2013.08.003**

## Introduction

The genus *Felis* is thought to have emerged close to 6.2 million years ago, during the late Miocene epoch [1]. Genetically, domestic cats are indistinguishable from the African wildcat, *Felis sylvestris lybica*, and archaeological evidence confirms that the close relationship between cats and humans was evident nearly 10 000 years ago in the Middle East [2]. The *Felis* lineage has suffered invasions by viruses from three subfamilies of the *retroviridae*; the *spumavirus* FeFV (feline foamy virus), the *gammaretrovirus* FeLV (feline leukaemia virus), and the *lentivirus* FIV (feline immunodeficiency virus). Lentiviruses are endemic in Felidae species (reviewed in [3]) and appear to have invaded species such as the American puma (*P. concolor*) many thousands of years ago, prior to the spread of pumas from South America to North America (10–12 000 years ago, after the last ice age). In contrast, the absence of lentiviruses from the majority of species closely related to the domestic cat suggests a recent introduction and spread subsequent to domestication. The relatively recent spread of FIV through the domestic cat population and the striking similarities between the pathogenesis of FIV in cats, and of AIDS in HIV-infected individuals offers a unique opportunity to compare the evolution of the host–virus relationship and the development of immunity to infection.

FIV targets CD4+ helper T cells *via* an initial high affinity interaction between Env and CD134 (OX40) [4^••^,5^•^] and a subsequent interaction with the chemokine receptor CXCR4 [6,7]. Expression of CD134 in the cat is restricted largely to activated CD4+ T cells [8^•^]. Accordingly, FIV infection of the cat results in a progressive depletion of CD4+ helper T cells and the development of an AIDS-like immune dysfunction. The ensuing immunodeficiency manifests with chronic gingivitis and stomatitis, anorexia, cachexia, neurological signs and an increased incidence of malignancy.

**Figure 1:**
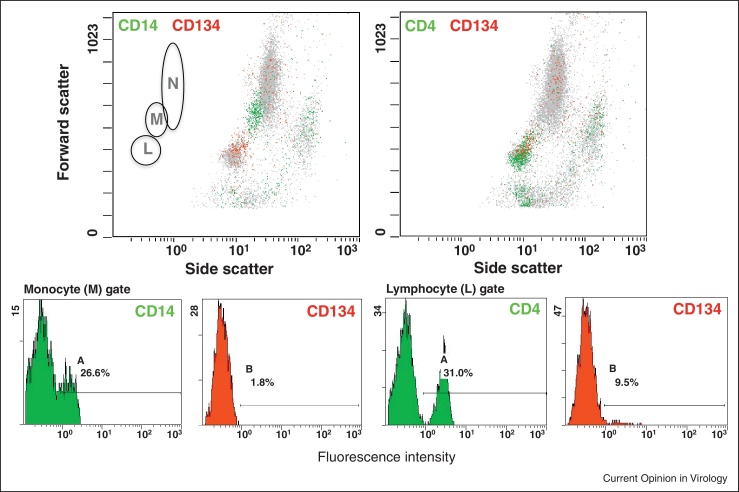
Restriction of feline CD134 expression to CD4+ T cells. CD134 expression was evaluated by flow cytometry on feline peripheral blood mononuclear cells, setting analysis gates for either lymphocytes (L), monocytes (M) or neutrophils (N). While CD14 expression was restricted primarily to the monocyte gate (upper left panel, green), CD134 expression (red) localised predominantly to the lymphocyte gate, coincident with the expression of CD4 (upper right panel). Additional CD134 expression was evident in the neutrophil analysis gate, consistent with the reported expression of CD134 on human neutrophils [43].

Although the selective targeting of FIV to helper T cells may be explained by the restricted expression pattern of CD134, in early infection, the primary cellular targets for the virus are not only CD4+ helper T cells, but also monocytes. Later in infection the tropism of the virus extends further to encompass both CD8+ T cells and B cells (reviewed in [9]). CD134 was first described as a surface antigen that was expressed almost exclusively on CD4+ T cells [10], thus the extended tropism of FIV during the course of infection would seem counter-intuitive. The development of antibody reagents with which the surface expression of feline CD134 may be measured, confirmed the expression of CD134 on feline CD4+ T cells [5^•^,8^•^] and monocyte-derived macrophages [8^•^], with lower levels of expression on a minor population of activated CD8+ T cells and on CD45R+ (B220) B cells [8^•^] (Figure 1). The up-regulation of CD134 on feline CD4+ T cells following activation [4^••^,5^•^], and the high levels of surface expression achieved [8^•^], are consistent with the selective targeting of these cells in early infection. While CD134 expression is ostensibly undetectable on feline peripheral blood (CD14+) monocytes, weak expression can be detected on splenic macrophages in culture and is upregulated following activation with lipopolysaccharide [8^•^]. Although CD134 is expressed at lower levels on macrophages than on activated CD4+ helper T cells, since macrophages are rich in CXCR4, a scenario may be envisaged whereby following initial infection and spread, the virus disseminates into compartments where CD134 expression is a limiting factor, principally cells of the monocyte/macrophage lineage, and as yet uncharacterised subpopulations of B220+ B cells and activated CD8+ T cells.

## Not all FIVs bind CD134 in the same way

**Figure 2:**
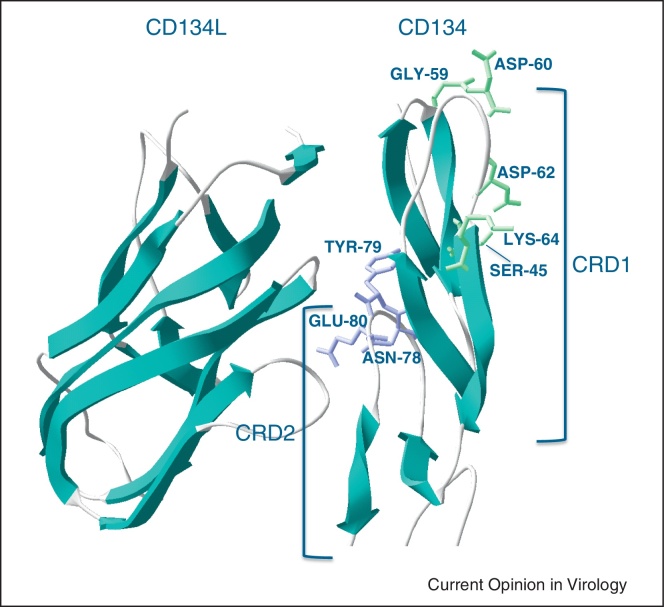
Residues critical to the function of feline CD134 as a receptor for FIV. Binding and syncytium formation by CRD2-independent strains is conferred upon human CD134 by inserting SER-45, GLY-59, ASP-60, ASP-62 and LYS-64 from CRD1 (shown in green) into human CD134. CRD2-dependent strains require additional determinants in CRD2, ASN-78, TYR-79 and GLU-80 (shown in purple) for the receptor to function. Structures were modelled by submitting feline CD134 and CD134L sequences (AB128982 and DQ269941) to the SWISS-MODEL automated protein modelling server [44].

The primary determinant of the FIV Env–CD134 interaction has been mapped to the first cysteine-rich domain (CRD1) of CD134 [11^••^,12^•^]. As human CD134 does not mediate FIV infection [4^••^] (one of the primary barriers to the transmission of FIV from cats to humans), chimeric CD134 molecules based on human CD134 and which bear the CRD1 of feline CD134 will support infection with selected strains of FIV [11^••^,12^•^]. Indeed, using this approach, a fully functional receptor for the PPR strain of FIV could be reconstituted by exchanging residues H45S, R59G, S60D, N62D, V64K in human CD134 [11^••^]. Importantly, well-characterised pathogenic strains of FIV, namely C-PGammer and GL8 (Glasgow-8) require additional determinants in the second cysteine-rich domain (CRD-2) of CD134 for infection [13^••^]. C-PGammer was derived by serial passage of plasma-borne virus during the acute phase of infection [14] while GL8 was isolated from a cat during a brief episode of illness during the protracted latent period of infection (subsequently the cat lived for a further eight years). Thus, these viruses are likely representative of viruses that dominate in early infection and, as such, may share properties with viruses that are transmitted between cats in nature. Accordingly, C-PGammer and GL8-like viruses may serve as biologically relevant challenge strains for trials of candidate vaccines. The amino acid motif 78NYE80 at the crown of a loop in CD134 CRD2 that participates in the interaction between CD134 and its cognate ligand (CD134L) is the essential determinant in CRD2 for CD134-usage by C-PGammer and GL8 (Figure 2). That GL8 and C-PGammer should be dependent upon residues distal to the projected Env binding face on CD134 is intriguing and may indicate that these viruses recognise a specific CD134 conformation that is sensitive to the CD134L–CD134 interaction [15^•^].

While the residues on CD134 that are important for FIV Env binding have been described [11^••^,12^•^,13^••^], the corresponding receptor binding determinants on Env are less well characterised. It is known that, with disease progression, viral variants emerge which infect host cells *via* a direct interaction with CXCR4 (CD134-independent infection). Critical determinants of CD134-independence reside in the V3-loop and single glutamate to lysine mutations in residues 407 and 409 of V3 promote CXCR4-dependent syncytium formation [16]. *In vivo*, there is a strong selective pressure to restore a glutamate residue at these positions and revert to virulence [16]. One of the driving forces for this reversion may be escape from neutralising antibodies induced by CD134-binding; a CD134-independent E407K mutant of PPR was readily susceptible to CD134-dependent neutralising antibodies [17].

## Evolution of CD134 usage *in vivo*

The virus–receptor interaction is a major determinant of the *in vivo* replicative capacity of HIV. For example, *ex vivo* analyses of primary HIV-1 isolates revealed that viral fitness mapped to the *env* gene [18,19]. Similarly, a comparison of the *envs* from two primary isolates of HIV-1 demonstrated that the reduced fitness associated with a clade C Env was associated with weak cell surface binding, inefficient entry, and an increased sensitivity to CCR5 antagonists and fusion inhibitors [20]. Moreover, HIV-1 clade B envelopes from early infection tend to harbour amino acid signatures that favour efficient expression of Env in infected cells, enhancing Env incorporation into nascent virions and facilitating replication to a high titre [21,22]. These signature sequences are lost during chronic infection under selective pressure from the adaptive immune response [21,22]. In this context, the requirement for the 78NYE80 motif within the CRD2 of CD134 for both C-PGammer and GL8 Envs may reflect a more efficient interaction between the viruses and their receptors. While infection with C-PGammer and GL8 is CRD2-dependent, strains such as PPR and B2542 have no such requirement. We postulated that CRD2-dependent infection might represent a feature of viruses that dominate ‘early’ in infection, facilitating both transmission and infection. Following establishment in the host, variants may then emerge that have a reduced requirement for an interaction with CRD2 of CD134, and in some cases may even lose the requirement for CRD2-binding altogether (as in PPR and B2542). This working hypothesis predicts that CRD2-independent viruses will be more prevalent in the ‘late’ stage of infection. In order to test this hypothesis, the receptor usage of viruses isolated six years post-infection with a clonal preparation of the CRD2-dependent GL8 strain was examined [23^•^]. While viral variants were identified that remained near identical to the challenge inoculum, additional variants were identified that displayed differences in the amino acid sequence of Env that switched the viral phenotype to CRD2-independent. Furthermore, these viruses displayed altered sensitivities to antagonists of the Env–CD134 interaction. While GL8 was markedly CRD2-dependent, resistant to anti-CD134 antibody and soluble CD134L (sCD134L) and sensitive to soluble CD134 (sCD134); variants that evolved *in vivo* were highly sensitive to anti-CD134 antibody, sensitive to sCD134L and resistant to sCD134 [23^•^]. That ‘late’ variants of GL8 should be sensitive to inhibition by anti-CD134 antibody is intriguing and may be significant, given that autoantibodies against CD134 have been detected in a proportion of FIV-infected cats [24^•^] and these autoantibodies were capable of blocking infection with the CRD2-independent PPR strain of FIV [24^•^]. Binding of the autoantibodies recognising CD134 in FIV infected cats required pre-engagement of CD134 with Env [24^•^], reminiscent of the polyreactive CD4-induced antibody 21c that recognises the HIV-1 Env–CD4 complex and binds determinants on both Env and CD4 [25]. Polyclonal B cell activation is a feature of both FIV and HIV infections and anti-CD4 autoantibodies have been well documented in HIV infection. Whether anti-CD134 and CD4 autoantibodies represent an anti-idiotypic response to antibodies raised against the viral Env protein or are epiphenomena related to the generalised immune dysregulation is unclear; nevertheless, the presence of such antibodies in the sera of FIV-infected cats may have the direct effect of suppressing the emergence of CRD2-independent viruses.

Engagement of CD4+ T cell-expressed CD134 by antigen presenting cell (APC)-expressed CD134L enhances the expansion and survival of antigen-specific CD4+ T cells (reviewed in [26]). Similarly, CD134L expressed on CD4+ T cells may contribute to the maintenance of CD4+ T cell longevity through T cell–T cell interactions [27]. Accordingly, the FIV–CD134 interaction may influence profoundly the development of antigen-specific immune responses as FIV will target preferentially the very cells that have been triggered to expand in response to infection, just as HIV infects HIV-specific CD4+ T cells preferentially [28]. Whether, APC-expressed CD134L, locally released soluble CD134 and CD134L, or anti-CD134 autoantibodies influence viral entry and expansion *in vivo* remains to be established. However, as the CD134L–CD134 interaction is a prerequisite for the expansion of antigen-specific CD4+ T cells, it would seem likely that the virus would have evolved to associate with the ligand bound form of CD134. Support for this model comes from structural studies indicating that the primary binding face for Env on CD134 is distinct from the CD134L-binding face mapped by X-ray crystallography [29^•^]. Accessibility of the FIV Env binding site on the ligand-engaged form of CD134 may facilitate the transmission of FIV bound to the surface of dendritic cells (through DC-SIGN [30]) to adjacent CD134-expressing CD4+ T cells. However, as FIV infection is modulated by engagement of CD134 by CD134L [15^•^], a scenario may also be envisaged in which the Env-CD134 interaction modulates signalling through CD134. CD134-engagement by CD134L recruits TNFR-associated factors (TRAFs) to the cytoplasmic tail of the molecule and triggers an NF-κB1-dependent signalling pathway; if native Env acts as a CD134 agonist, viral attachment to CD134 may promote the expansion and survival of the very target cells required for FIV replication. Conversely, if Env acts as an antagonist, attachment to CD134 may contribute to the selective depletion of FIV-specific CD4+ T cells.

## CD134-usage and transmission

If CD134 CRD2-dependent infection is a signature of FIV isolates from the ‘early’ stage of infection, such ‘early’ virus strains would appear to be the most appropriate targets for vaccine development. Assuming that both CRD2-dependent and CRD2-independent viruses are transmitted from an infected animal to a recipient during biting, what happens to CRD2-independent viruses following transmission? Are CRD2-independent viruses filtered from the inoculum during the process of transmission, analogous to the filtering of X4 variants of HIV-1 as articulated in the ‘gatekeeper’ hypothesis [31,32]? To resolve this question, a synthetic FIV quasispecies was prepared comprising matched doses of six clonal variants, identical in *gag* and *pol* but with unique *envs* derived from either CRD2-dependent or independent variants of FIV GL8. Following experimental transmission of this reconstituted quasispecies to naive animals, a selective expansion of CRD2-dependent viruses was observed [33^••^]. CRD2-independent viruses failed to thrive *in vivo*, achieving lower proviral loads. Further, the failure of the CRD2-independent viruses to thrive was independent of early neutralising antibody or cytotoxic T cell responses [33^••^]. These data suggest that CRD2-dependent viruses are able to replicate more efficiently in the host. Thus, clear parallels exist between the dominance of CCR5-dependent strains of HIV-1 and CD134 CRD2-dependent strains of FIV during early infection, culminating in the efficient targeting of a specific CD4+ helper T cell population.

## What drives the emergence of CD134 CRD2-independent viruses?

The Fel-O-Vax FIV vaccine (Pfizer Inc.) combines inactivated virus and inactivated cells with a proprietary adjuvant. At the core of the vaccine is the FL4 strain of FIV, a derivative of the prototypic (and CRD2-independent) Petaluma isolate of FIV. The Env of FL4 is curious in that it lacks two potential sites for N-linked glycosylation that are highly conserved amongst the majority of field strains of virus. The T271I and N342Y mutations in FL4 lie in a loop region that is similar in location to the V1V2 loop of HIV. When identical mutations were incorporated into the Envs of either GL8 or C-PGammer, receptor usage was switched from CRD2-dependent to CRD2-independent [34^•^]. Given the role of N-linked glycosylation in shielding Env from the humoral immune response, the humoral response to infection may offer a driving force for the switch from CRD2-dependent to CRD2-independent infection. Consistent with this hypothesis, viral variants that emerged *in vivo* following infection with a clonal preparation of GL8, and which resisted neutralisation by homologous serum, displayed a shift in receptor usage from CRD2-dependent to CRD-2 independent [23^•^]. For one variant, exchange of the hypervariable V5 loop of Env alone was sufficient to render the virus both neutralising antibody resistant and CRD2-independent [23^•^]. As polyclonal sera elicited in infected cats can be remarkably mono-specific, targeting single determinants in the variable loops of the virus [35,36], *in vivo* escape from neutralisation may be sufficient to drive the emergence of CRD2-independent viruses. In doing so, the progeny viruses may gain the ability to spread into additional cellular compartments through a direct (CD134-independent) interaction with CXCR4 [17].

## Conclusions

The replication of FIV *in vivo* may be influenced by both host and viral factors; in this review we have focussed on the Env–CD134 interaction. However, replication may also be influenced by additional viral determinants; transcriptional regulation by the long terminal repeats (LTRs) and the accessory gene *orfA* [37–41] and the ability of the virus to evade intrinsic immune factors such as APOBEC proteins [38,42]. Given that the virus–receptor interaction is the initial event in retroviral replication, understanding the intricacies of the Env–CD134 relationship will inform future strategies for the development of both FIV vaccines and therapeutics. Moreover, as the lentiviruses of cats and humans have evolved two distinct strategies to selectively target infection of CD4+ helper T cells, inter-species comparisons offer a unique opportunity to discern what makes immunodeficiency-causing lentiviruses tick.

## References and recommended reading

Papers of particular interest, published within the period of review, have been highlighted as:

• of special interest
•• of outstanding interest
